# Anti-CD40 predominates over anti-CTLA-4 to provide enhanced antitumor response of DC-CIK cells in renal cell carcinoma

**DOI:** 10.3389/fimmu.2022.925633

**Published:** 2022-08-25

**Authors:** Ying Zhang, Xiaolong Wu, Amit Sharma, Hans Weiher, Matthias Schmid, Glen Kristiansen, Ingo G. H. Schmidt-Wolf

**Affiliations:** ^1^ Department of Integrated Oncology, Center for Integrated Oncology (CIO), University Hospital Bonn, Bonn, Germany; ^2^ Department of Neurosurgery, University Hospital Bonn, Bonn, Germany; ^3^ Department of Applied Natural Sciences, Bonn-Rhein-Sieg University of Applied Sciences, Rheinbach, Germany; ^4^ Institute for Medical Biometry, Computer Science and Epidemiology, University Hospital Bonn, Bonn, Germany; ^5^ Institute of Pathology, University Hospital Bonn, Bonn, Germany

**Keywords:** cytokine-induced killer cells, immunotherapy, renal cell carcinoma, CD40, CTLA-4

## Abstract

Cytokine-induced killer cells (CIK) in combination with dendritic cells (DCs) have shown favorable outcomes in renal cell carcinoma (RCC), yet some patients exhibit recurrence or no response to this therapy. In a broader perspective, enhancing the antitumor response of DC-CIK cells may help to address this issue. Considering this, herein, we investigated the effect of anti-CD40 and anti-CTLA-4 antibodies on the antitumor response of DC-CIK cells against RCC cell lines. Our analysis showed that, a) anti-CD40 antibody (G28.5) increased the CD3+CD56+ effector cells of CIK cells by promoting the maturation and activation of DCs, b) G28.5 also increased CTLA-4 expression in CIK cells *via* DCs, but the increase could be hindered by the CTLA-4 inhibitor (ipilimumab), c) adding ipilimumab was also able to significantly increase the proportion of CD3+CD56+ cells in DC-CIK cells, d) anti-CD40 antibodies predominated over anti-CTLA-4 antibodies for cytotoxicity, apoptotic effect and IFN-γ secretion of DC-CIK cells against RCC cells, e) after ipilimumab treatment, the population of Tregs in CIK cells remained unaffected, but ipilimumab combined with G28.5 significantly reduced the expression of CD28 in CIK cells. Taken together, we suggest that the agonistic anti-CD40 antibody rather than CTLA-4 inhibitor may improve the antitumor response of DC-CIK cells, particularly in RCC. In addition, we pointed towards the yet to be known contribution of CD28 in the crosstalk between anti-CTLA-4 and CIK cells.

## Introduction

Among immunotherapies, cytokine-induced killer (CIK) cell therapy holds a significant place, as evidenced by several completed or ongoing clinical trials, which also included 15 clinical trials in renal cell carcinoma (RCC) (1). For patients diagnosed with inoperable or metastatic RCC, systemic therapies, including immunotherapy, are typically adopted (2). CIK cell therapy has exhibited promising clinical effects on those patients, but recurrence and non-responsiveness is still a challenging issue. Therefore, several efforts are currently underway to improve the antitumor response of CIK cells (3). Of interest, an International Registry of CIK Cells (IRCC) has been established to summarize the results of clinical trials using CIK cells (4).

CIK cells represent a heterogeneous population of exceptional T lymphocytes with CD3+CD56+ cells as the primary effectors (5, 6). CIK cells possess both non-MHC-bound cytotoxicity and antitumor activity of T lymphocytes (7). Importantly, as dendritic cells (DCs) are the most efficient antigen-presenting cells (APCs), the combination of DCs and CIK cells has shown a significant increase in cytotoxic activity (8). CD40 is a member of the tumor necrosis factor (TNF) receptor superfamily and mainly expressed on B cells, DCs, monocytes, and endothelial cells (9, 10). According to one study, an agonistic anti-CD40 antibody (CP-870,893) stimulated DCs and further promoted the antitumor response of lymph node-derived T cells (11). Among other determinants, CTLA-4 is also known as a negative regulator of T cells (12). CTLA-4 is primarily expressed in the intracellular vesicles of T cells and functions by outcompeting CD28 in binding CD80/CD86 on APCs, or enhancing the activity of regulatory T cells (Tregs) (13, 14). A phase I study reported that the combination of CP-870,893 and a CTLA-4-blocking mAb (tremelimumab) resulted in T-cell resuscitation and caused tolerable toxicity in metastatic melanoma (15).

As aforementioned, CIK cells have shown promising clinical efficacy and safety in RCC (16–18). Besides, DCs pulsed with tumor lysate cocultured with CIK cells or activated simultaneously with pembrolizumab (PD-1 inhibitor) have shown favorable antitumor response in RCC patients (19–21). Considering this, herein, we sought to enhance the antitumor response of DC-CIK cells, presumably as an option for unresponsive patients. To achieve this, we activated DC-CIK cells with anti-CD40 and anti-CTLA-4 antibodies in RCC cell lines. In addition, we investigated the cytotoxicity potential, early/late apoptosis levels, and IFN-γ secretion levels. Moreover, we assessed the population of Tregs in CIK cells and highlighted the yet to be known CTLA-4-CD28 interaction in this spectrum.

## Materials and methods

### Cell lines and antibodies

We utilized two human renal carcinoma cell lines Caki-2 and ACHN which were purchased from CLS Cell Lines Service GmbH (Eppelheim, Germany). Both were maintained in RPMI-1640 medium (PAN-Biotech, Aidenbach, Germany) supplemented with 10% fetal bovine serum (FBS, Gibco, USA), 100 U/ml penicillin, and 100 μg/ml streptomycin (Gibco, Grand Island, NY, USA) at 37°C, 5% CO_2_. Prior to experiments, the cell lines were controlled with MycoAlert mycoplasma detection kit (Lonza, Basel, Switzerland).

Concerning antibodies, mouse anti-human CD40 antibody (clone G28.5) was purchased from Bio X Cell (Lebanon, NH, USA). The anti-CTLA-4 antibody ipilimumab was purchased from Selleckchem (Houston, TX, USA). The mouse IgG1 (mIgG1, isotype control of G28.5) and human IgG1 (hIgG1, isotype control of ipilimumab) isotype control were also purchased from Bio X Cell. The following fluorescent monoclonal antibodies and their isotype controls were purchased from BioLegend (San Diego, CA): mouse anti-human CD3-FITC (OKT3), mouse anti-human CD8a-Brilliant Violet 421 (RPA-T8), mouse anti-human CD56-PE (5.1H11), mouse anti-human CD154-APC (22–29), mouse anti-human CD152-APC (L3D10), mouse anti-human CD28-PerCP/Cyanine5.5 (CD28.2), mouse anti-human CD14-FITC (M5E2), mouse anti-human CD16-FITC (3G8), mouse anti-human CD19-FITC (SJ25C1), mouse anti-human CD20-FITC (2H7), mouse anti-human CD56-FITC (HCD56), mouse anti-human HLA-DR-PE (L243), mouse anti-human CD11c-PerCP/Cyanine5.5 (S-HCL-3), mouse anti-human CD40-APC (5C3), mouse anti-human CD80-APC (2D10), mouse anti-human CD83-APC (HB15e), mouse anti-human CD86-APC (BU63), mouse anti-human CD3-PerCP/Cyanine5.5 (OKT3), mouse anti-human CD4-APC (OKT4), mouse anti-human CD25-PE (BC96), mouse anti-human CD127-FITC (A019D5).

### Generation of DCs

To generate DCs, peripheral blood mononuclear cells (PBMCs) were isolated from buffy coats of healthy donors (University Hospital Bonn) as previously described (30). As next, PBMCs were set to 5 × 10^6^/ml in complete medium (RPMI-1640 medium supplemented with 10% FBS, 100 U/ml penicillin, 100 μg/ml streptomycin, and 2.5% HEPES Buffer 1M (PAN-Biotech, Aidenbach, Germany) and allowed to adhere to 6-well plates for 3 h, 37°C. Subsequently, we aspirated the medium containing non-adherent cells and washed with warm medium to remove the non-adherent cells. The adherent cells were further cultured with 1000 U/ml GM-CSF and 1000 U/ml IL-4 (ImmunoTools, Friesoythe, Germany) in complete medium to generate DCs. The medium with necessary cytokines was replaced every 2-3 days. To prepare tumor lysate, ACHN and Caki-2 cells were digested with 0.05% Trypsin-EDTA (Gibco, USA) and washed thrice with PBS. Pelleted cells were resuspended in PBS and lysed *via* five freeze-thaw cycles. The cell lysate was then centrifuged for 10 min, 13500 g at 4°C. The supernatant was collected and sterilized through a 0.22 μm filter membrane. The protein concentration of the water-soluble tumor lysate was determined using Pierce BCA Protein-Assay kit (Thermo Scientific, USA) and stored at −80°C. After six days of culture, the obtained tumor lysate was added to DC culture medium to load DCs at the concentration of 100 μg/ml for 48h. Also,1000 U/ml TNF-α (ImmunoTools, Friesoythe, Germany) was added to promote maturation for another 24 h.

### DC-CIK cell coculture and phenotype analysis

CIK cells were generated as previously described (31). The non-adherent cells were collected and stimulated by 1000 U/mL IFN-γ (ImmunoTools, Friesoythe, Germany) for 24 h. On day 1, 50 ng/mL anti-CD3 monoclonal antibody (eBioscience, Thermo Fisher Scientific, San Diego, CA, USA), 600 U/mL IL-2 (ImmunoTools, Friesoythe, Germany), 100 U/ml IL-1β (ImmunoTools, Friesoythe, Germany) were added to continue inducing CIK cells. Cells were then incubated at 37°C in a humidified atmosphere of 5% CO_2_. Fresh medium with 600 U/mL IL-2 was replenished every 2-3 days. In ipilimumab or isotype control group, ipilimumab or human IgG1 was added in CIK cells only once at a concentration of 10 μg/ml at the initiation of culture. After nine days of culture, DCs were collected and cocultured with CIK cells at a ratio of 1:5 in complete medium supplemented with 600 U/ml IL-2. DC-CIK cells were harvested after 2-3 days for further experiments.

For phenotype analysis, DCs and CIK cells were washed and resuspended in 100 μl FACS buffer at the concentration of 1 × 10^7^ cells/ml. DCs were stained with PE-HLA-DR, PerCP/Cyanine 5.5-CD11c, APC-CD40, APC-CD80, APC-CD83, APC-CD86, FITC-CD3, FITC-CD14, FITC-CD16, FITC-CD19, FITC-CD20, and FITC-CD56 on ice for 20 min in the dark. For phenotyping of CIK cells, FITC-CD3, Brilliant Violet 421-CD8a, PE-CD56, APC-CD40L, APC-CTLA-4, and PerCP/Cyanine5.5-CD28 were used to stain. To detect Tregs, the cells were incubated with PerCP/Cyanine5.5-CD3, APC-CD4, Brilliant Violet 421-CD8a, PE-CD25, and FITC-CD127. After two washings, cells were then stained with Zombie Aqua Fixable Viability Kit (BioLegend, San Diego, CA) to exclude dead cells. Samples were acquired on FACSCanto II (BD Bioscience). Notably, the intracellular expression of CTLA-4 was detected according to manufacturer’s instruction. Briefly, CIK cells were fixed with 100 μl Fixation buffer (Invitrogen, Waltham, MA, USA) for 30 min at room temperature. After two subsequent washings by 2 ml 1 × Permeabilization Buffer (Invitrogen, Waltham, MA, USA), cells were resuspended in 100 μl 1 × Permeabilization Buffer and incubated with APC-CTLA-4 for 40 min in the dark. The cells were subsequently washed by 2 ml 1 × Permeabilization Buffer, 2 ml PBS, and resuspended for analysis.

### Generation of activated T cells

Activated T cells were generated as described by Yano et al. (22). Briefly, PBMCs were activated with 20 ng/mL anti-CD3 monoclonal antibody and expanded with 100 U/ml IL-2 in complete medium for 14 days. 10 μg/ml ipilimumab or human IgG1 was added to CIK cells only once at the initiation of culture.

### Cell counting kit-8 (CCK-8) assay

CCK-8 was performed to test the direct effect of G28.5 and ipilimumab on tumor cells. ACHN (1 × 10^4^ cells/well) and Caki-2 (0.5 × 10^4^ cells/well) cells were incubated with 1 µg/ml, 10 µg/ml, 20 µg/ml, and 40 µg/ml of G28.5 and ipilimumab in 100 μl total volume in 96-well plates. After 24 h of coculture, 10 μl CCK-8 solution (Dojindo, Japan) was added to each well and incubated with cells for about 3h. The absorbance at 450 nm was measured on Multiskan GO Microplate Spectrophotometer (Thermo Scientific). The viability of tumor cells was calculated using the following formula: Viability = (OD experimental - OD blank)/(OD control − OD blank) × 100%.

### Cytotoxicity assay

The cytotoxicity of DC-CIK cells against RCC cells was detected using flow cytometry, as previously described (31). ACHN and Caki-2 cells were labeled by CellTrace™ Violet dye (Invitrogen, Waltham, MA, USA) and distributed in 96-well plates at 3 × 10^4^ cells/well and 1.2 × 10^4^ cells/well, respectively. DC-CIK cells, ipilimumab, or hIgG1 treated DC-CIK cells were then cocultured with tumor cells at an E/T ratio of 10:1. After being incubated with 10 μg/ml G28.5 or mIgG1 control for 24 h, all cells were collected, and 7-aminoactinomycin D (7-AAD) was added for live and dead cell discrimination by flow cytometry. The formula used for cytotoxicity calculation is as follows: Cytotoxicity = ((CL − TL)/CL) × 100%. CL, percentage of live tumor cells in control tubes (tumor cells alone); TL, percentage of live tumor cells in test tubes (treatment groups).

### Apoptosis assay

Apoptosis was measured according to the protocol described for FITC Annexin V Apoptosis Detection Kit with 7-AAD (BioLegend). Briefly, the tumor cells were labeled by CellTrace™ Violet dye, and cocultured with DC-CIK cells, ipilimumab, or hIgG1 treated DC-CIK cells at an E/T ratio of 5:1. After incubation with 10 μg/ml G28.5 or mIgG1 control for 10-14 h, all cells were collected and resuspended in 100 μl Annexin V binding buffer. Five microliter FITC Annexin V and 7-AAD were added and incubated at room temperature for 15 min in the dark. Another 200 μl Annexin V binding buffer was added to each tube, and samples were then analyzed by flow cytometry.

### ELISA assay

The IL-12 secretion of DCs was detected by the IL-12 p70 Human ELISA Kit (Invitrogen). First, DCs were distributed at 1 × 10^6^ cells/well in 24-well plates in the presence or absence of 10 μg/ml G28.5, ipilimumab, and their isotype controls mIgG1 and hIgG1. After 24h, the supernatant was collected to detect IL-12 secretion by ELISA. The IFN-γ secretion was also detected according to manufacturer’s instruction. DC-CIK cells, ipilimumab, or hIgG1 treated DC-CIK cells were cocultured with tumor cells (5 × 10^4^ cells/well) at a ratio of 10:1. Subsequently, the cells were incubated with 10 μg/ml G28.5 or mIgG1 control for 24 h. The supernatant was collected to determine the concentration of IFN-γ by the IFN gamma Human ELISA Kit (Invitrogen).

### Statistical analysis

FACS data sets were analyzed using FlowJo v10.6 software (FlowJo, LLC, Ashland, Oregon, U.S.A.). Statistical analyses were performed using GraphPad Prism v.8.0 (GraphPad Software, Inc., San Diego, CA, U.S.A.). Quantitative data are presented as means ± SD. Differences between groups were investigated using Student’s unpaired and paired t-tests, and one-way ANOVA with a Bonferroni correction for multiple comparisons. Each experiment was performed in triplicates and repeated at least three times. *P* < 0.05 was considered statistically significant.

## Results

### Characterizing the phenotype of CIK cells and DCs

**Figure 1:**
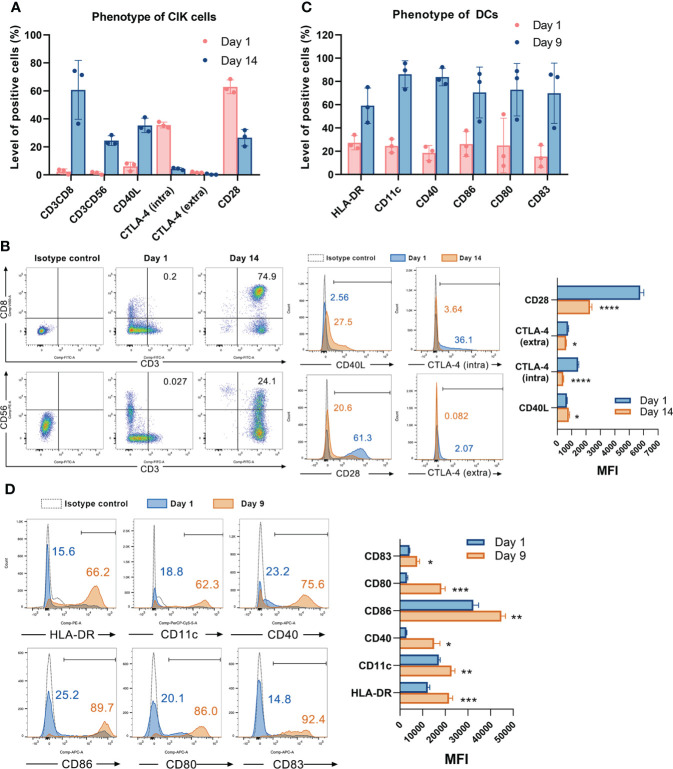
Characterizing the phenotype of CIK cells and DCs. **(A)** The phenotype of CIK cells on day 1 and day 14 was detected by flow cytometry. Each bar represents mean ± SD of three donors. One representative phenotyping analysis of CIK cells and quantification of the mean fluorescence intensity (MFI) are shown in **(B)**. **(C)** The phenotype of DCs on day 1 and day 9 was detected by flow cytometry. Each bar represents mean ± SD of three donors. One representative phenotyping analysis of DCs and quantification of the MFI are shown in **(D)**. Intra: intracellular; extra: extracellular; SD: standard deviation. (* *P* < 0.05, ** *P* < 0.01, *** *P* < 0.001, **** *P* < 0.0001).

We first determined the phenotype of CIK cells and DCs by flow cytometry. CD3+CD56+ and CD3+CD8+ cells, which primarily contribute to the cytotoxicity of CIK cells, were found to be significantly increased by 23.2 ± 2.1% (*P* = 0.0004) and 58.4 ± 12.2% (*P* = 0.0088), individually after 14 days of culture (**Figures 1A, B**). In addition, the level of cells positive for CD40L (the ligand of CD40) was also elevated from 6.0 ± 3.0% to 35.3 ± 5.2% (*P* = 0.0011). Notably, the CTLA-4 (especially the intracellular CTLA-4) positive cells was significantly decreased from 35.6 ± 2.1% to 4.3 ± 0.8% (*P* < 0.0001). Of interest, its counterpart CD28+ cells were also significantly reduced from 62.9 ± 5.0% to 26.57 ± 5.8% (*P* = 0.0012). Similarly, **Figure 1B** showed that the mean fluorescence intensity (MFI) of CD40L was increased from 671.7 ± 26.5 to 799.3 ± 61.2 (*P* = 0.0294), whereas the MFI of CD28, intracellular and extracellular CTLA-4 were decreased from 5762.0 ± 249.5, 1449.3 ± 47.7, and 744.0 ± 49.4 to 2270.3 ± 136.6 (*P* < 0.0001), 419.7 ± 28.1 (*P* < 0.0001), and 616.0 ± 19.3 (*P* = 0.0139), respectively. Following nine days of generation, the mature DCs showed significantly increased expression of HLA-DR, CD11c, CD40, CD86, CD80, and CD83 (**Figures 1C, D**). Gating strategy for CIK cells and DCs was shown in **Figure S1**.

### Anti-CD40 antibodies increased CD3+CD56+ population of CIK cells *via* DCs

**Figure 2:**
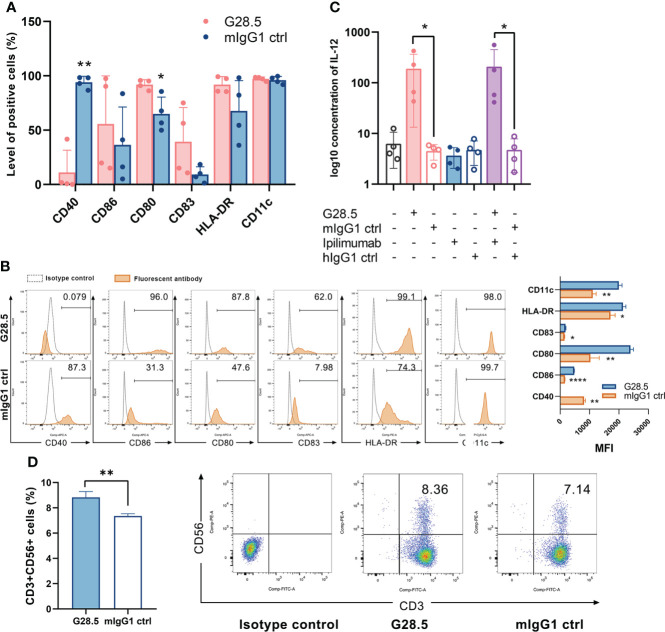
G28.5 increases the CD3+CD56+ population of CIK cells by inducing DC maturation and activation. **(A)** The anti-CD40 antibody G28.5 improved DC mature phenotype. DCs on day 6 were plated at 25 × 10^4^ cells/ml in the presence of G28.5 (10 μg/ml) or its isotype control mIgG1 (10 μg/ml) for 48 h. After treatment, phenotypic markers of DCs were detected by flow cytometry. Each bar represents mean ± SD of four donors. One representative phenotyping analysis and quantification of the MFI are shown in **(B)**. **(C)** G28.5 promoted the IL-12 secretion of DCs. DCs were distributed at 1 × 10^6^ cells/well in 24-well plates in the presence or absence of 10 μg/ml G28.5, ipilimumab, and their isotype controls mIgG1 and hIgG1. After 24 h, the supernatant was collected to detect IL-12 secretion by ELISA. Each bar represents mean ± SD of four donors. **(D)** G28.5 increased the CD3+CD56+ population of CIK cells when cocultured with DCs. CIK cells on day 9 were labeled by CellTrace™ Violet dye and cocultured with DCs at a ratio of 5:1. DC-CIK cells and CIK cells were treated with 10 μg/ml G28.5 or mIgG1 for 48 h. The expression of CD3 and CD56 on CIK cells was measured by flow cytometry. One representative donor (left) and the flow cytometry analysis (right) are shown. (* *P* < 0.05, ** *P* < 0.01, **** P < 0.0001).

Next, we incubated DCs with anti-CD40 antibody (G28.5) for 48 hours and observed upregulation of CD80, CD83, C86, and HLA-DR, hence showing the maturation of DCs (**Figures 2A, B**). Hunter et al. observed similar results when a different anti-CD40 antibody (CP-870,893) was used (11). G28.5 also significantly stimulated the secretion of IL-12 compared to control by 185.6 ± 79.9 pg/ml (*P* = 0.046) and 204.1 ± 79.9 pg/ml (*P* = 0.046) in G28.5 and the combination group, individually, indicating the enhanced activation of DCs following G28.5 treatment (**Figure 2C**). We further treated DC-CIK and CIK cells with G28.5 to assess any alteration in the primary effector CD3+CD56+ cells. An increased CD3+CD56+ population of CIK cells was observed in DC-CIK cells alone (*P* = 0.007) (**Figures 2D**, **S2A**), indicating that G28.5 could promote the antitumor response of CIK cells by inducing DC maturation and activation.

### Favorable effects of G28.5 combined with ipilimumab on CTLA-4 and CD3+CD56+ effector cells

**Figure 3:**
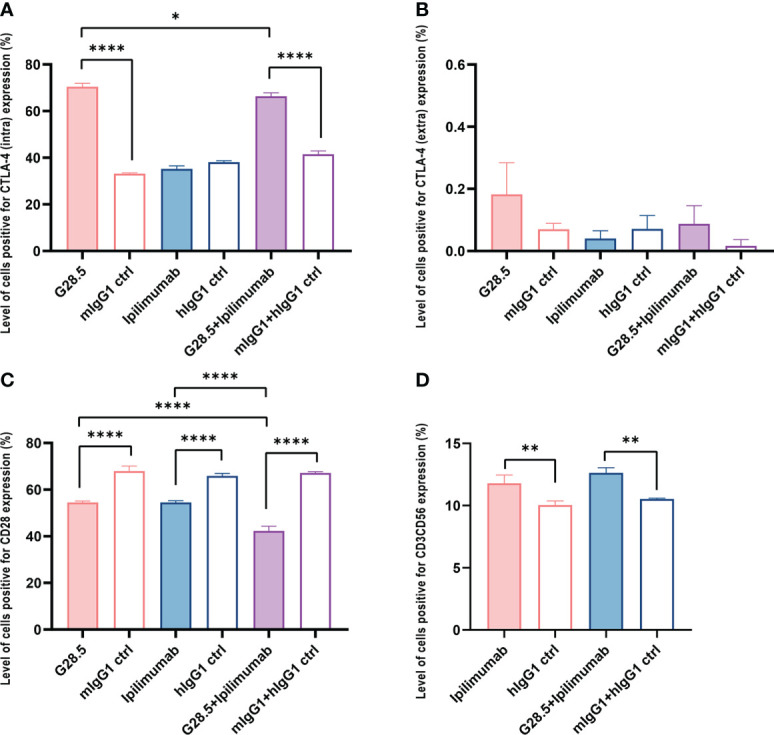
G28.5 and ipilimumab have different effects on the CTLA-4 expression of CIK cells. CIK cells treated with 10 μg/ml ipilimumab or hIgG1 for nine days or not were labeled by CellTrace™ Violet dye and cocultured with DCs at a ratio of 5:1. DC-CIK cells and CIK cells were treated with 10 μg/ml G28.5 or mIgG1 for another 48 h. The expression of intracellular and extracellular markers was detected by flow cytometry. Each bar represents mean ± SD of one representative donor. **(A)** The effect of G28.5 and ipilimumab on intracellular expression of CTLA-4. **(B)** The effect of G28.5 and ipilimumab on extracellular expression of CTLA-4. **(C)** The effect of G28.5 and ipilimumab on the expression of CD28. **(D)** Ipilimumab increased the CD3+CD56+ population of CIK cells. Intra: intracellular; extra: extracellular. (* *P* < 0.05, ** *P* < 0.01, **** *P* < 0.0001).

We next evaluated the effect of G28.5 in combination with ipilimumab (CTLA-4 inhibitor) on DC-CIK cells, following 48 hours of incubation. We found that G28.5 increased intracellular and extracellular expression of the CTLA-4 by 37.3 ± 0.9% (*P* < 0.0001) and 0.1 ± 0.1% (*P* = 0.24), respectively in CIK cells when cocultured with DCs, but not in CIK cells alone (**Figures 3A, B**, **S2B, C**). In addition, ipilimumab suppressed the CTLA-4 expression of CIK cells that had been promoted by G28.5 in DC-CIK cells (P = 0.0203). Hence, it suggested that G28.5 combined with ipilimumab might have a favorable effect on the antitumor response. Moreover, G28.5 and ipilimumab both decreased the expression of CD28 (the counterpart of CTLA-4) in DC-CIK cells. The level of cells positive for CD28 were reduced by 13.5 ± 1.3% (*P* < 0.0001) by G28.5 and 11.4 ± 0.7% (*P* < 0.0001) by ipilimumab, respectively, and by 24.9 ± 1.2% (*P* < 0.0001) by the combination in DC-CIK cells (**Figure 3C**). The MFI of CD28 was also decreased by G28.5 and ipilimumab accordingly (**Figure S3**). However, G28.5 had no direct effect on CD28 expression of CIK cells alone (**Figure S2D**). Similar to G28.5, ipilimumab increased the CD3+CD56+ population in CIK cells alone (1.8 ± 0.2%, *P* = 0.001) or DC-CIK cells (1.8 ± 0.4%, *P* = 0.006) (**Figures 3D**, **S2E**). The combination of G28.5 and ipilimumab also increased the percentage of CD3+CD56+ cells in DC-CIK cells compared with G28.5 (by 3.8 ± 0.3%, *P* < 0.0001) and ipilimumab (by 0.8 ± 0.3%, *P* = 0.3742). Overall, it can be assumed that G28.5 in combination with ipilimumab could increase the antitumor efficacy of DC-CIK cells compared to G28 or ipilimumab alone, primarily by reducing inhibitory CTLA-4 and proliferating CD3+CD56+ effector cells.

### Assessing the direct effect of anti-CD40, anti-CTLA-4 and DC-CIK cells on RCC cell lines

**Figure 4:**
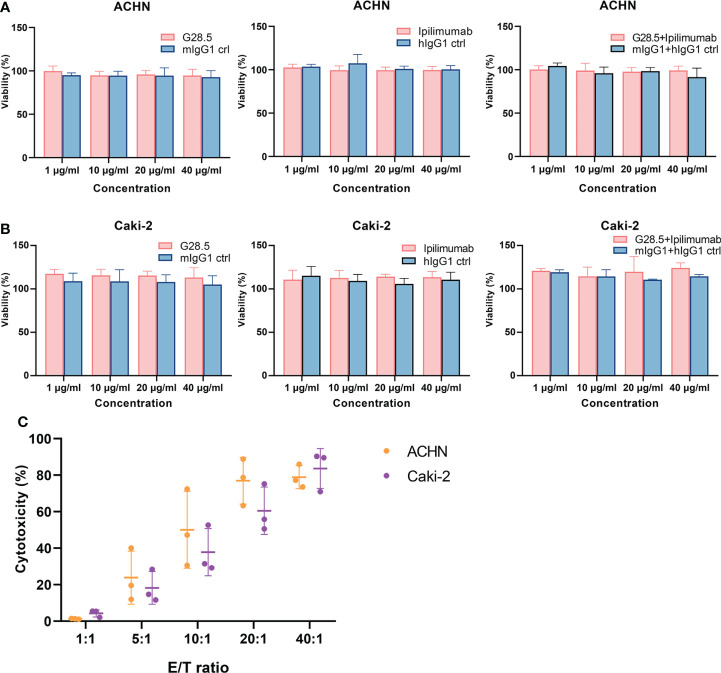
Assessing the direct effect of anti-CD40, anti-CTLA-4 and DC-CIK cells on RCC cell lines. **(A)** No direct effect was observed on G28.5 and ipilimumab against ACHN. ACHN cells were distributed in 96-well plates at 1 × 10^4^ cells/well. G28.5 (left), ipilimumab (middle), the combination (right) and their isotype controls were added at different concentrations (1μg/ml, 10μg/ml, 20μg/ml, and 40μg/ml) for 24 h. Data are expressed as mean ± SD of one representative experiment. **(B)** No direct effect was observed on G28.5 and ipilimumab against Caki-2. Caki-2 cells were distributed in 96-well plates at 0.5 × 10^4^ cells/well and treated as **(A)**. **(C)** DC-CIK cells are cytotoxic against ACHN and Caki-2. ACHN (blue) and Caki-2 (red) cells were labeled by CellTrace™ Violet dye to distinguish them from DC-CIK cells. Tumor cells were distributed in 96-well plates and incubated 24 hours with DC-CIK cells at the E/T ratio of 1:1, 5:1, 10:1, 20:1, and 40:1. Then all cells were collected, and 7-AAD was added to detect dead cells by flow cytometry. Each bar represents mean ± SD of three donors.

We next sought to exclude any direct effect of G28.5 and ipilimumab on RCC cell lines (ACHN and Caki-2 cells). Our CCK-8 assay showed no apparent growth inhibition in ACHN cells after incubation with G28.5, ipilimumab, or the combination compared to isotype controls (**Figure 4A**). The same was observed on Caki-2 cells (**Figure 4B**). In addition, we tested the cytotoxicity of DC-CIK cells on RCC cells using flow cytometry (**Figure 4C**). To mention, ACHN and Caki-2 cells were first labeled with CellTrace™ Violet dye so that they could be distinguished from DC-CIK cells. When tumor cells were cocultured with DC-CIK cells at varying E/T ratios (1:1, 5:1, 10:1, 20:1, 40:1), we found that at E/T ratios above 5:1, DC-CIK cells efficiently killed ACHN and Caki-2 cells with cytotoxicity ranging from 20% to 90%. The gating strategy for analysis of cytotoxicity was shown in **Figure S4**.

### Anti-CD40 antibody enhanced cytotoxicity, apoptotic effect and IFN-γ secretion of DC-CIK cells

**Figure 5:**
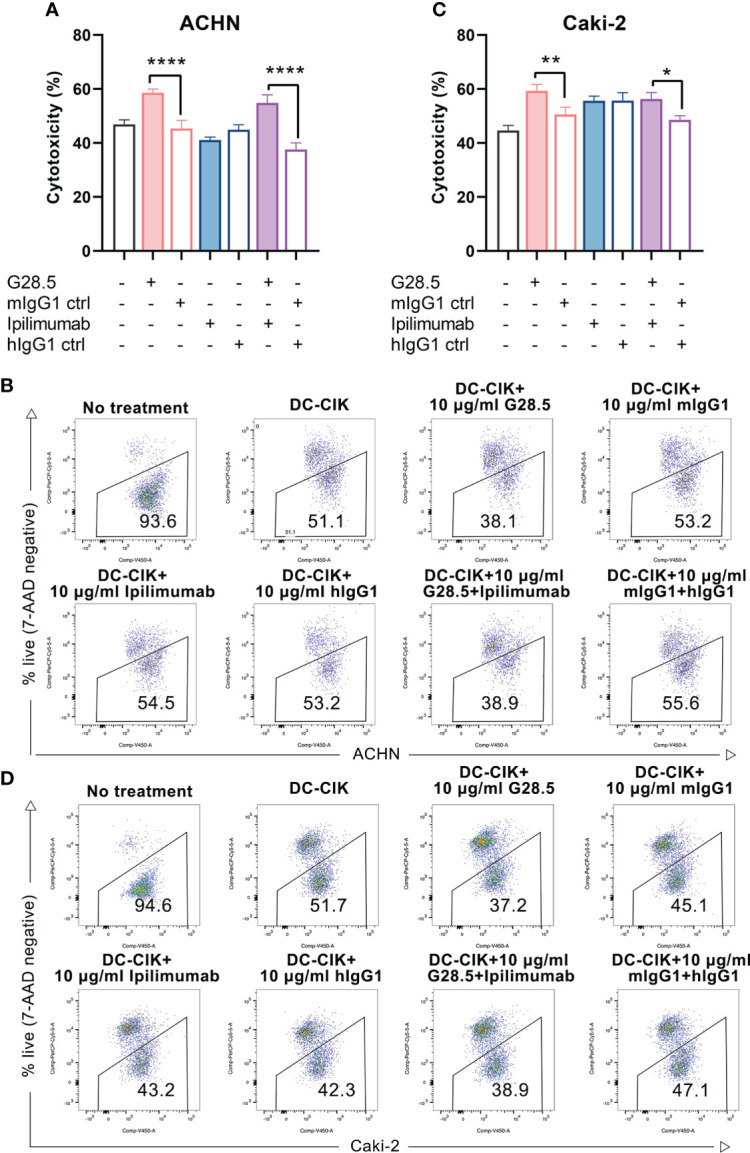
Cytotoxicity of DC-CIK cells treated with G28.5 and ipilimumab against RCC cell lines. **(A)** Cytotoxicity of DC-CIK cells treated with G28.5 or ipilimumab against ACHN. ACHN cells were labeled by CellTrace™ Violet dye and distributed in 96-well plates at 3 × 10^4^ cells/well. DC-CIK cells, ipilimumab or hIgG1 treated DC-CIK cells were then cocultured with ACHN cells at an E/T ratio of 10:1. After the incubation with 10 μg/ml G28.5 or mIgG1 control for 24 h, all cells were collected, and 7-AAD was added to detect the dead cells by flow cytometry. Each bar represents mean ± SD of one representative donor. One representative flow cytometry analysis is shown in **(B)**. **(C)** Cytotoxicity of DC-CIK cells treated with G28.5 or ipilimumab against Caki-2 cells. Caki-2 cells were labeled by CellTrace™ Violet dye and distributed in 96-well plates at 1.2 × 10^4^ cells/well. The following procedures were performed as above. One representative flow cytometry analysis is shown in **(D)**. (* *P* < 0.05, ** *P* < 0.01, **** *P* < 0.0001).

Considering the above assumption that G28.5 in combination with ipilimumab could promote the antitumor response of DC-CIK cells, we cocultured DC-CIK cells with CellTrace™ Violet-labeled tumor cells at an E/T ratio of 10:1. We found that G28.5 significantly increased the cytotoxicity of DC-CIK cells in ACHN by 13.2 ± 1.8% (*P* < 0.0001) in the G28.5 group and 17.3 ± 1.8% (*P* < 0.0001) in the combination group (**Figures 5A, B**). Similar results were observed in the case of Caki-2, where G28.5 increased the cytotoxicity of DC-CIK cells by 8.7 ± 1.9% (*P* = 0.007), and in combination by 7.7 ± 1.9% (*P* = 0.021) (**Figures 5C, D**). In contrast, the anti-CTLA-4 antibody ipilimumab did not increase the antitumor activity of DC-CIK cells in any cell lines.

**Figure 6:**
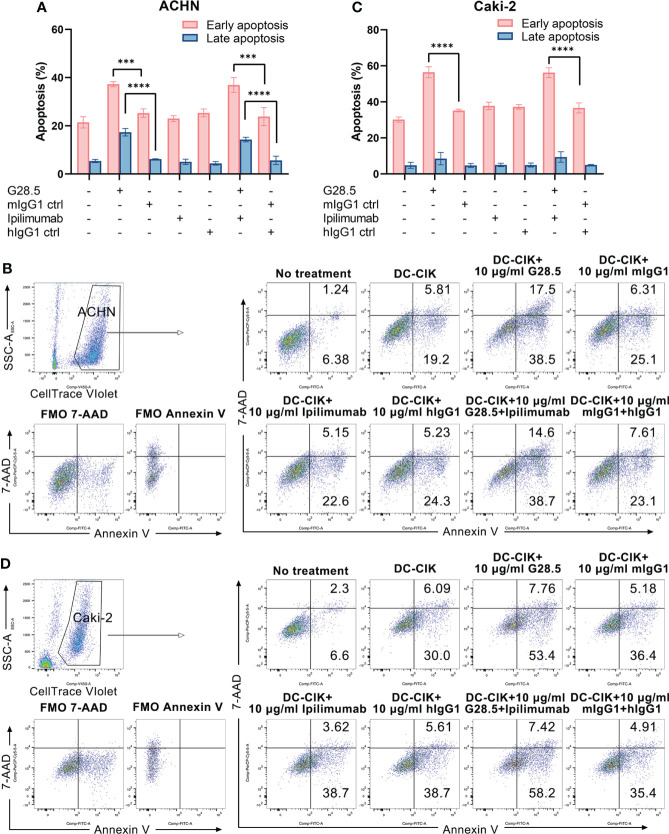
Apoptosis of RCC cells treated with DC-CIK cells combined with G28.5 and ipilimumab. **(A)** Early and late apoptosis of ACHN cells treated with DC-CIK cells in combination with G28.5 and ipilimumab. ACHN cells were labeled by CellTrace™ Violet dye and distributed in 96-well plates at 3 × 10^4^ cells/well. Tumor cells were cocultured with DC-CIK cells, ipilimumab or hIgG1 treated DC-CIK cells at an E/T ratio of 5:1. After incubation with 10 μg/ml G28.5 or mIgG1 control for 10-14 h, Annexin V-FITC/7-AAD was used to detect the early (Annexin V+7-AAD-) and late apoptosis (Annexin V+7-AAD+) by flow cytometry. Each bar represents mean ± SD of one representative donor. One representative flow cytometry analysis is shown in **(B)**. **(C)** Early and late apoptosis of Caki-2 cells treated with DC-CIK cells in combination with G28.5 and ipilimumab. Caki-2 cells were labeled by CellTrace™ Violet dye and distributed in 96-well plates at 1.2 × 10^4^ cells/well. The following procedures were performed as **(A)**. One representative flow cytometry analysis is shown in **(D)**. (*** *P* < 0.001, **** *P* < 0.0001).

Additionally, we investigated whether apoptosis of RCC cells can be increased by DC-CIK cells using G28.5 and ipilimumab. To mention, we detected both early and late apoptosis using Annexin V-FITC/7-AAD. In G28.5 alone group, early apoptosis of ACHN was significantly increased by 12.0 ± 1.9% (*P* = 0.0004), while by 13.1 ± 1.9% (*P* = 0.0001) in combination with ipilimumab (**Figures 6A, B**). The enhanced effect on late apoptosis was also observed for G28.5 (by 11.2 ± 0.9%, *P* < 0.0001) and in combination (by 8.7 ± 0.9%, *P* < 0.0001). Similar to ACHN cells, the early apoptosis of Caki-2 was significantly increased in both G28.5 group (by 21.3 ± 1.8%, P < 0.0001) and the combination group (by 19.6 ± 1.8%, *P* < 0.0001) (**Figures 6C, D**). Here again, anti-CTLA-4 antibody ipilimumab did not increase the early or late apoptosis in any cell lines.

**Figure 7:**
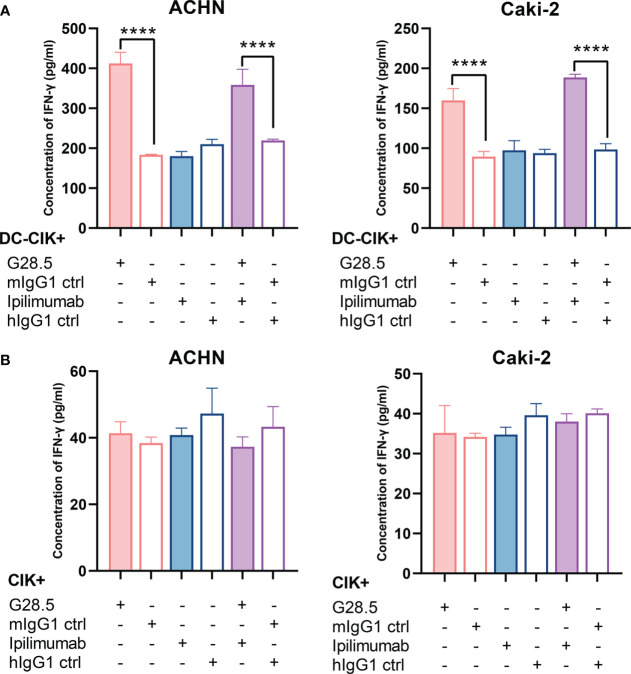
Effect of G28.5 and ipilimumab on IFN-γ secretion of DC-CIK and CIK cells. **(A)** The IFN-γ secretion of DC-CIK cells against ACHN (left) and Caki-2 (right) cells. DC-CIK cells, ipilimumab or hIgG1 treated DC-CIK cells were cocultured with tumor cells (5 × 10^4^ cells/well) at a ratio of 10:1. Cell were incubated with 10 μg/ml G28.5 or mIgG1 control for 24 h. At the end of incubation, the supernatant was collected to determine the concentration of IFN-γ by ELISA. Each bar represents mean ± SD of one representative donor. **(B)** The IFN-γ secretion of CIK cells against ACHN (left) and Caki-2 (right) cells. (**** *P* < 0.0001).

We next investigated whether these two antibodies could promote the secretion of IFN-γ, a cytokine required for tumor killing by NK and cytotoxic T cells. We found that G28.5 used alone or in combination with ipilimumab significantly increased the IFN-γ secretion from DC-CIK cells against ACHN (by 228.6 ± 17.06 pg/ml, *P* < 0.0001 and 139.1 pg/ml ± 17.06, *P* < 0.0001, respectively), compared to the isotype control (**Figure 7A**). Similar results were found in case of Caki-2 cells (by 70.3 ± 7.4 pg/ml, *P* < 0.0001 and 90.1 ± 7.4 pg/ml, *P* < 0.0001, respectively). The anti-CTLA-4 antibody ipilimumab showed no effect on IFN-γ levels in any cell line. Considering the peculiar function of DC-CIK cells compared to CIK cells, we supplemented G28.5 in CIK cells (instead of DC-CIK cells) and failed to detect any increased IFN-γ level (**Figure 7B**). This suggested that G28.5 could enhance the antitumor response of CIK cells solely by promoting the maturation and activation of DCs.

### Ipilimumab did not alter the Treg population in CIK cells

**Figure 8:**
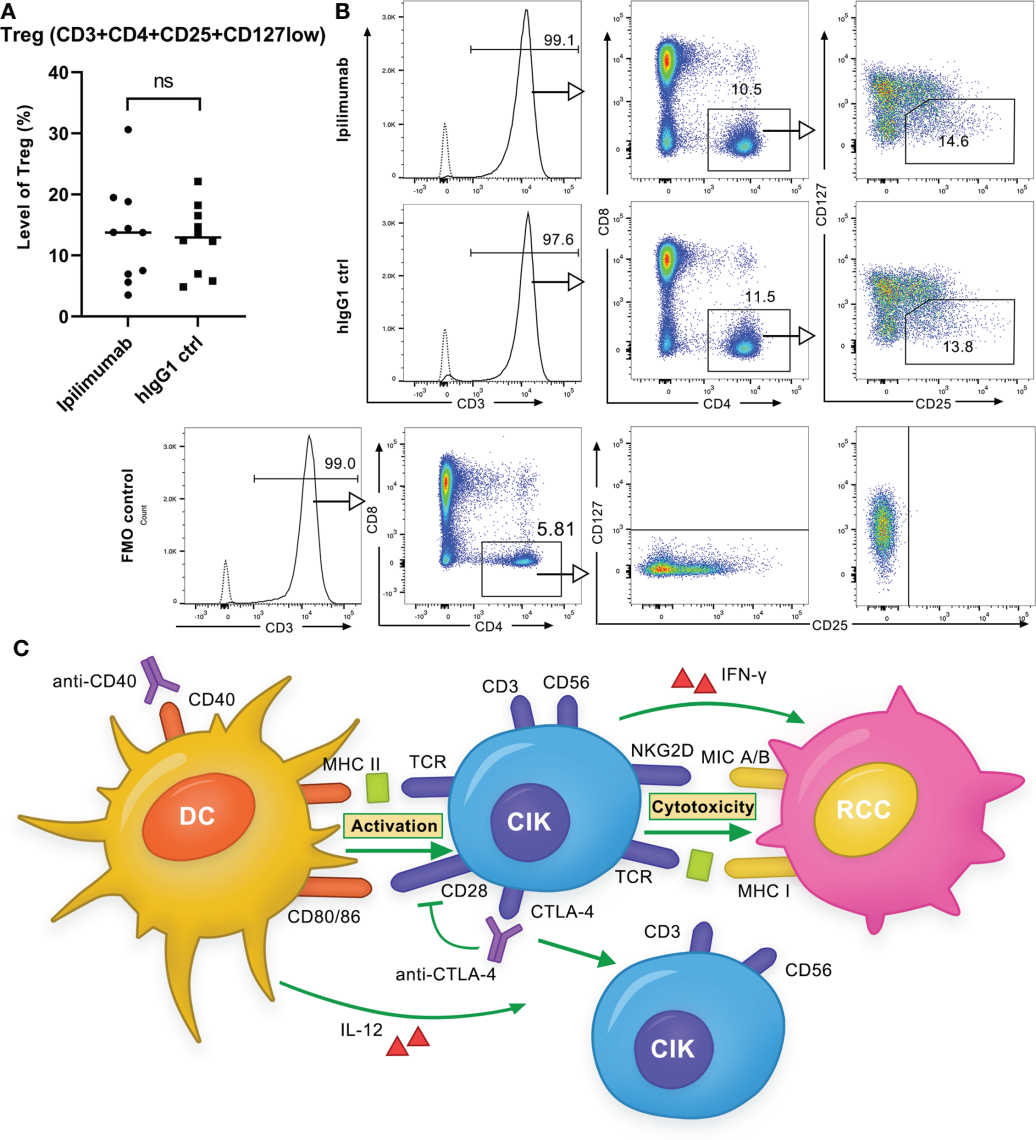
Ipilimumab is ineffective in decreasing the Treg population in CIK cells. **(A)** Treg population in CIK cells after treatment with ipilimumab or isotype control. 10 μg/ml ipilimumab or hIgG1 control was added to CIK cells only once at the initiation of culture. Tregs (CD3 + CD4 + CD25 + CD127low) in CIK cells of ten donors were detected by flow cytometry. Each bar represents the mean ± SD of ten donors. (*ns* not significant) **(B)** One representative flow cytometry analysis is shown. **(C)** Schematic of the effect of anti-CD40 and anti-CTLA-4 antibodies on DC-CIK cells. Anti-CD40 antibody activated DCs increased the cytotoxicity of CIK cells against RCC cells, whereas the anti-CTLA-4 antibody had a very restrictive effect on the antitumor response. The anti-CTLA-4 antibody increased the proportion of CD3+CD56+ effector cells but decreased CD28 expression.

As we did not observe an enhancement in antitumor response of DC-CIK cells by ipilimumab, we sought to investigate whether another independent determinant (e.g., Tregs) may be a contributing factor. Since, it has been discussed that tumor regression caused by anti-CTLA-4 antibodies may rely on a selective reduction of Tregs but not checkpoint blockade (23, 24). We therefore compared the population of Tregs in CIK cells after ipilimumab treatment, as described previously (22). Similar to the results of Yano et al., after 14-day incubation of activated T cells with 10 μg/ml ipilimumab, Tregs (CD3+CD4+CD25+CD127low) were found to be significantly reduced by 4.2 ± 2.3% (*P* = 0.034) in activated T cells compared to the controls (**Figure S5**). Besides, a decrease in the CD4/CD8 ratio was also observed (*P* = 0.326). In contrast, when CIK cells were incubated with 10 μg/ml ipilimumab, the proportion of Tregs remained unaffected (*P* = 0.567) (**Figures 8A, B**). This may partially explain why the addition of ipilimumab did not enhance the antitumor response of DC-CIK cells. The hypothesized mechanism of anti-CD40 and anti-CTLA-4 antibodies on DC-CIK cells is shown in **Figure 8C**.

## Discussion

Immunotherapy has become a feasible treatment option for cancer patients, still a fraction among them remains unresponsive towards the standard treatment regimen. Among cancer therapies, CIK cell therapy has proven to be successful, as evident from the numerous clinical trials. CIK cells have been licensed in various countries, including Germany. But considering the non-responsive patients, several efforts are being performed to make this approach more efficient. One convenient option that is worth considering is to promote the antitumor response of CIK/DC-CIK cells. Recent studies also provided insights into the enhancement of their cytotoxicity (25, 26). In the current manuscript, we sought to investigate whether the combination of anti-CD40 and anti-CTLA-4 antibodies can considerably enhance the antitumor response of DC-CIK cells against RCC cell lines. To achieve this, we first characterize the phenotype of CIK cells and DCs, and observed that the anti-CD40 antibody (G28.5) increased the CD3+CD56+ population of CIK cells *via* DCs. In addition, we found that the antitumor response of DC-CIK cells was significantly improved by G28.5, while the CTLA-4 inhibitor ipilimumab showed a very restrictive effect.

Since, it is well established that tumor cell escape often occurs through the impaired antigen recognition by the immune system, or the establishment of an immunosuppressive state in tumor microenvironment (e.g., Tregs) (27). We therefore sought to enhance the tumor-killing activity of DC-CIK cells in two distinct ways: First, by promoting maturation and activation of antigen-presenting DCs through CD40 ligation, and second, by suppressing inhibitory signaling in the effector cells or reducing Tregs by CTLA-4 blockade. In this context, it has been previously shown that the combination of anti-CD40 and anti-CTLA-4 antibodies accompanying a liposomal peptide or adenoviral vaccine considerably enhanced the CTL responses against tumor cells (28, 29). And CD40 activation on DC cells has previously been demonstrated to increase Th1 cell proliferation (32). In our analysis, we first found the possibility that G28.5 in combination with ipilimumab might increase the antitumor efficacy of DC-CIK cells compared to G28.5 or ipilimumab alone, primarily by reducing inhibitory CTLA-4 signaling and proliferating CD3+CD56+ effector cells. However, our results showed that anti-CD40 antibody predominated over anti-CTLA-4 antibody for cytotoxicity, apoptotic effect and IFN-γ secretion of DC-CIK cells against RCC cells. To mention, despite some clinical differences in the RCC cell lines (Caki-2: male/69 years, clear cell renal cell carcinoma; ACHN: male/22 years, renal cell adenocarcinoma), our results were consistent in both of them. Therefore, the possibility of heterogeneity between these cancer cell lines (in addition to genetic-epigenetic variations) as a confounding factor can be excluded, as discussed by Sharma et al. (33).

To determine the potential reason for ipilimumab inefficacy, we further investigated whether another independent factor (e.g., Tregs) might play a role. For instance, it has been discussed that tumor regression caused by anti-CTLA-4 antibodies may rely on a selective reduction of Tregs (23, 24). We therefore compared the population of Tregs in CIK cells after ipilimumab treatment, but no changes were found. However, we noticed that both G28.5 and ipilimumab significantly reduced the expression of CD28 in CIK cells. It is well known that CD28 has a similar structure to CTLA-4 but has an opposite effect on T cell immunity. CD28-mediated co-signaling is related to cytokine production and T cell proliferation (12). In context to our data, how this decrease in CD28 accounts for the crosstalk between ipilimumab and CIK cells needs further investigation. Notably, the expression of CTLA-4 decreased sharply after two weeks of culture, suggesting that the inhibitory signaling of CTLA-4 may not play an essential role in the cytotoxicity of CIK cells. It is worth mentioning that, a) whether these observed changes are RCC specific or also occur in other cancers needs to be further investigated; b) translating our results to *in vivo* models will also provide more information about the contribution of tumor microenvironment.

## Conclusion

To summarize, our data suggest that the agonistic anti-CD40 antibody rather than CTLA-4 inhibitor may improve the antitumor response of DC-CIK cells, particularly in RCC. In addition, we pointed towards the yet to be known contribution of CD28 in the crosstalk between anti-CTLA-4 and CIK cells.

## Data availability statement

The raw data supporting the conclusions of this article will be made available by the authors, without undue reservation.

## Ethics statement

Ethical review and approval was not required for the study on human participants in accordance with the local legislation and institutional requirements. Written informed consent for participation was not required for this study in accordance with the national legislation and the institutional requirements.

## Author contributions

Conceptualization: YZ and IS-W. Methodology: YZ. Investigation: YZ and XW. Validation: YZ, XW, and AS. Formal analysis: YZ, XW, and AS. Data curation: YZ and IS-W. Writing – original draft preparation: YZ. Writing – review and editing: IS-W, AS, HW, MS, GK, and XW. Visualization: YZ and AS. Supervision: IS-W, HW, MS, and GK. Project administration: IS-W. All authors contributed to the article and approved the submitted version.

## Acknowledgments

CIO is kindly supported by Deutsche Krebshilfe (DKH), Bonn, Germany.

## Conflict of interest

The authors declare that the research was conducted in the absence of any commercial or financial relationships that could be construed as a potential conflict of interest.

## Publisher’s note

All claims expressed in this article are solely those of the authors and do not necessarily represent those of their affiliated organizations, or those of the publisher, the editors and the reviewers. Any product that may be evaluated in this article, or claim that may be made by its manufacturer, is not guaranteed or endorsed by the publisher.

## Abbreviations

CIK cell, cytokine-induced killer cell; RCC, renal cell carcinoma; IRCC, International Registry of CIK Cells; APC, antigen-presenting cell; TNF, tumor necrosis factor; PBMC, peripheral blood mononuclear cell; CCK-8, cell counting kit-8; 7-AAD, 7-aminoactinomycin D.

## References

[1] Zhang Y Ellinger J Ritter M Schmidt-Wolf IGH. Clinical studies applying cytokine-induced killer cells for the treatment of renal cell carcinoma. Cancers (Basel) (2020) 12(9):2471. doi: 10.3390/cancers12092471 PMC7564072 32882824

[2] Hsieh JJ Purdue MP Signoretti S Swanton C Albiges L Schmidinger M. Renal cell carcinoma. Nat Rev Dis Primers (2017) 3:17009. doi: 10.1038/nrdp.2017.9 28276433 PMC5936048

[3] Sharma A Schmidt-Wolf IGH. 30 years of cik cell therapy: Recapitulating the key breakthroughs and future perspective. J Exp Clin Cancer Res CR (2021) 40(1):388. doi: 10.1186/s13046-021-02184-2 34886895 PMC8662881

[4] Zhang Y Schmidt-Wolf IGH. Ten-year update of the international registry on cytokine-induced killer cells in cancer immunotherapy. J Cell Physiol (2020) 235(12):9291–303. doi: 10.1002/jcp.29827 32484595

[5] Schmidt-Wolf IGH Lefterova P Mehta BA Fernandez LP Huhn D Blume KG. Phenotypic characterization and identification of effector cells involved in tumor cell recognition of cytokine-induced killer cells. Exp Hematol (1993) 21(13):1673–9. 7694868

[6] Gütgemann S Frank S Strehl J Schmidt-Wolf IGH. Cytokine-induced killer cells are type ii natural killer T cells. German Med Sci GMS e-journal (2007) 5:Doc07. PMC2703238 19675715

[7] Introna M. Cik as therapeutic agents against tumors. J Autoimmun (2017) 85:32–44. doi: 10.1016/j.jaut.2017.06.008 28679475

[8] Wang QJ Wang H Pan K Li YQ Huang LX Chen SP. Comparative study on anti-tumor immune response of autologous cytokine-induced killer (Cik) cells, dendritic cells-cik (Dc-cik), and semi-allogeneic dc-cik. Chin J Cancer (2010) 29(7):641–8. doi: 10.5732/cjc.009.10772 20591215

[9] Armitage RJ Fanslow WC Strockbine L Sato TA Clifford KN Macduff BM. Molecular and biological characterization of a murine ligand for Cd40. Nature (1992) 357(6373):80–2. doi: 10.1038/357080a0 1374165

[10] Banchereau J Bazan F Blanchard D Brière F Galizzi JP van Kooten C. The Cd40 antigen and its ligand. Annu Rev Immunol (1994) 12:881–922. doi: 10.1146/annurev.iy.12.040194.004313 7516669

[11] Hunter TB Alsarraj M Gladue RP Bedian V Antonia SJ. An agonist antibody specific for Cd40 induces dendritic cell maturation and promotes autologous anti-tumour T-cell responses in an *in vitro* mixed autologous tumour Cell/Lymph node cell model. Scand J Immunol (2007) 65(5):479–86. doi: 10.1111/j.1365-3083.2007.01927.x 17444959

[12] Rudd CE Taylor A Schneider H. Cd28 and ctla-4 coreceptor expression and signal transduction. Immunol Rev (2009) 229(1):12–26. doi: 10.1111/j.1600-065X.2009.00770.x 19426212 PMC4186963

[13] Peggs KS Quezada SA Chambers CA Korman AJ Allison JP. Blockade of ctla-4 on both effector and regulatory T cell compartments contributes to the antitumor activity of anti-Ctla-4 antibodies. J Exp Med (2009) 206(8):1717–25. doi: 10.1084/jem.20082492 PMC2722174 19581407

[14] Schneider H Mandelbrot DA Greenwald RJ Ng F Lechler R Sharpe AH. Cutting edge: Ctla-4 (Cd152) differentially regulates mitogen-activated protein kinases (Extracellular signal-regulated kinase and c-jun n-terminal kinase) in Cd4+ T cells from Receptor/Ligand-deficient mice. J Immunol (Baltimore Md 1950) (2002) 169(7):3475–9. doi: 10.4049/jimmunol.169.7.3475 12244135

[15] Bajor DL Mick R Riese MJ Huang AC Sullivan B Richman LP. Long-term outcomes of a phase I study of agonist Cd40 antibody and ctla-4 blockade in patients with metastatic melanoma. Oncoimmunology (2018) 7(10):e1468956. doi: 10.1080/2162402x.2018.1468956 30288340 PMC6169575

[16] Wang Z Liu X Till B Sun M Li X Gao Q. Combination of cytokine-induced killer cells and programmed cell death-1 blockade works synergistically to enhance therapeutic efficacy in metastatic renal cell carcinoma and non-small cell lung cancer. Front Immunol (2018) 9:1513. doi: 10.3389/fimmu.2018.01513 30026742 PMC6041387

[17] Wang Z Zhang Y Liu Y Wang L Zhao L Yang T. Association of myeloid-derived suppressor cells and efficacy of cytokine-induced killer cell immunotherapy in metastatic renal cell carcinoma patients. J Immunother (2014) 37(1):43–50. doi: 10.1097/cji.0000000000000005 24316555

[18] Zhang J Zhu L Wei J Liu L Yin Y Gu Y. The effects of cytokine-induced killer cells for the treatment of patients with solid tumors: A clinical retrospective study. J Cancer Res Clin Oncol (2012) 138(6):1057–62. doi: 10.1007/s00432-012-1179-1 PMC11824252 22392076

[19] Zhan HL Gao X Pu XY Li W Li ZJ Zhou XF. A randomized controlled trial of postoperative tumor lysate-pulsed dendritic cells and cytokine-induced killer cells immunotherapy in patients with localized and locally advanced renal cell carcinoma. Chin Med J (2012) 125(21):3771–7. 23106871

[20] Zhao X Zhang Z Li H Huang J Yang S Xie T. Cytokine induced killer cell-based immunotherapies in patients with different stages of renal cell carcinoma. Cancer Lett (2015) 362(2):192–8. doi: 10.1016/j.canlet.2015.03.043 25843292

[21] Chen CL Pan QZ Weng DS Xie CM Zhao JJ Chen MS. Safety and activity of pd-1 blockade-activated dc-cik cells in patients with advanced solid tumors. Oncoimmunology (2018) 7(4):e1417721. doi: 10.1080/2162402x.2017.1417721 29632736 PMC5889206

[22] Yano H Thakur A Tomaszewski EN Choi M Deol A Lum LG. Ipilimumab augments antitumor activity of bispecific antibody-armed T cells. J Trans Med (2014) 12:191. doi: 10.1186/1479-5876-12-191 PMC4105782 25008236

[23] Selby MJ Engelhardt JJ Quigley M Henning KA Chen T Srinivasan M. Anti-Ctla-4 antibodies of Igg2a isotype enhance antitumor activity through reduction of intratumoral regulatory T cells. Cancer Immunol Res (2013) 1(1):32–42. doi: 10.1158/2326-6066.Cir-13-0013 24777248

[24] Du X Tang F Liu M Su J Zhang Y Wu W. A reappraisal of ctla-4 checkpoint blockade in cancer immunotherapy. Cell Res (2018) 28(4):416–32. doi: 10.1038/s41422-018-0011-0 PMC5939050 29472691

[25] Yu SJ Ma C Heinrich B Brown ZJ Sandhu M Zhang Q. Targeting the crosstalk between cytokine-induced killer cells and myeloid-derived suppressor cells in hepatocellular carcinoma. J Hepatol (2019) 70(3):449–57. doi: 10.1016/j.jhep.2018.10.040 PMC6380944 30414862

[26] Wu X Sharma A Oldenburg J Weiher H Essler M Skowasch D. Nkg2d engagement alone is sufficient to activate cytokine-induced killer cells while 2b4 only provides limited coactivation. Front Immunol (2021) 12. doi: 10.3389/fimmu.2021.731767 PMC8529192 34691037

[27] Schreiber RD Old LJ Smyth MJ. Cancer immunoediting: Integrating immunity's roles in cancer suppression and promotion. Science (2011) 331(6024):1565–70. doi: 10.1126/science.1203486 21436444

[28] Ito D Ogasawara K Iwabuchi K Inuyama Y Onoé K. Induction of ctl responses by simultaneous administration of liposomal peptide vaccine with anti-Cd40 and anti-Ctla-4 mab. J Immunol (2000) 164(3):1230–5. doi: 10.4049/jimmunol.164.3.1230 10640735

[29] Sorensen MR Holst PJ Steffensen MA Christensen JP Thomsen AR. Adenoviral vaccination combined with Cd40 stimulation and ctla-4 blockage can lead to complete tumor regression in a murine melanoma model. Vaccine (2010) 28(41):6757–64. doi: 10.1016/j.vaccine.2010.07.066 20682365

[30] Schmidt-Wolf IGH Negrin RS Kiem HP Blume KG Weissman IL. Use of a scid Mouse/Human lymphoma model to evaluate cytokine-induced killer cells with potent antitumor cell activity. J Exp Med (1991) 174(1):139–49. doi: 10.1084/jem.174.1.139 PMC2118875 1711560

[31] Wu X Zhang Y Li Y Schmidt-Wolf IGH. Increase of antitumoral effects of cytokine-induced killer cells by antibody-mediated inhibition of mica shedding. Cancers (Basel) (2020) 12(7):1818. doi: 10.3390/cancers12071818 PMC7408690 32645836

[32] Fallarino F Grohmann U Vacca C Bianchi R Fioretti MC Puccetti P. Cd40 ligand and ctla-4 are reciprocally regulated in the Th1 cell proliferative response sustained by Cd8(+) dendritic cells. J Immunol (2002) 169(3):1182–8. doi: 10.4049/jimmunol.169.3.1182 12133938

[33] Sharma A Reutter H Ellinger J. DNA Methylation and bladder cancer: Where genotype does not predict phenotype. Curr Genomics (2020) 21(1):34–6. doi: 10.2174/1389202921666200102163422 PMC7324896 32655296

