# Supplementary material for: Anti-CD40 predominates over anti-CTLA-4 to provide enhanced antitumor response of DC-CIK cells in renal cell carcinoma
Source: Front Immunol. 2022 Aug 25;13:925633. doi: 10.3389/fimmu.2022.925633 (PMC9453234; doi:10.3389/fimmu.2022.925633)
Supplement: Supplementary file 1 [file DataSheet_1.docx]

**
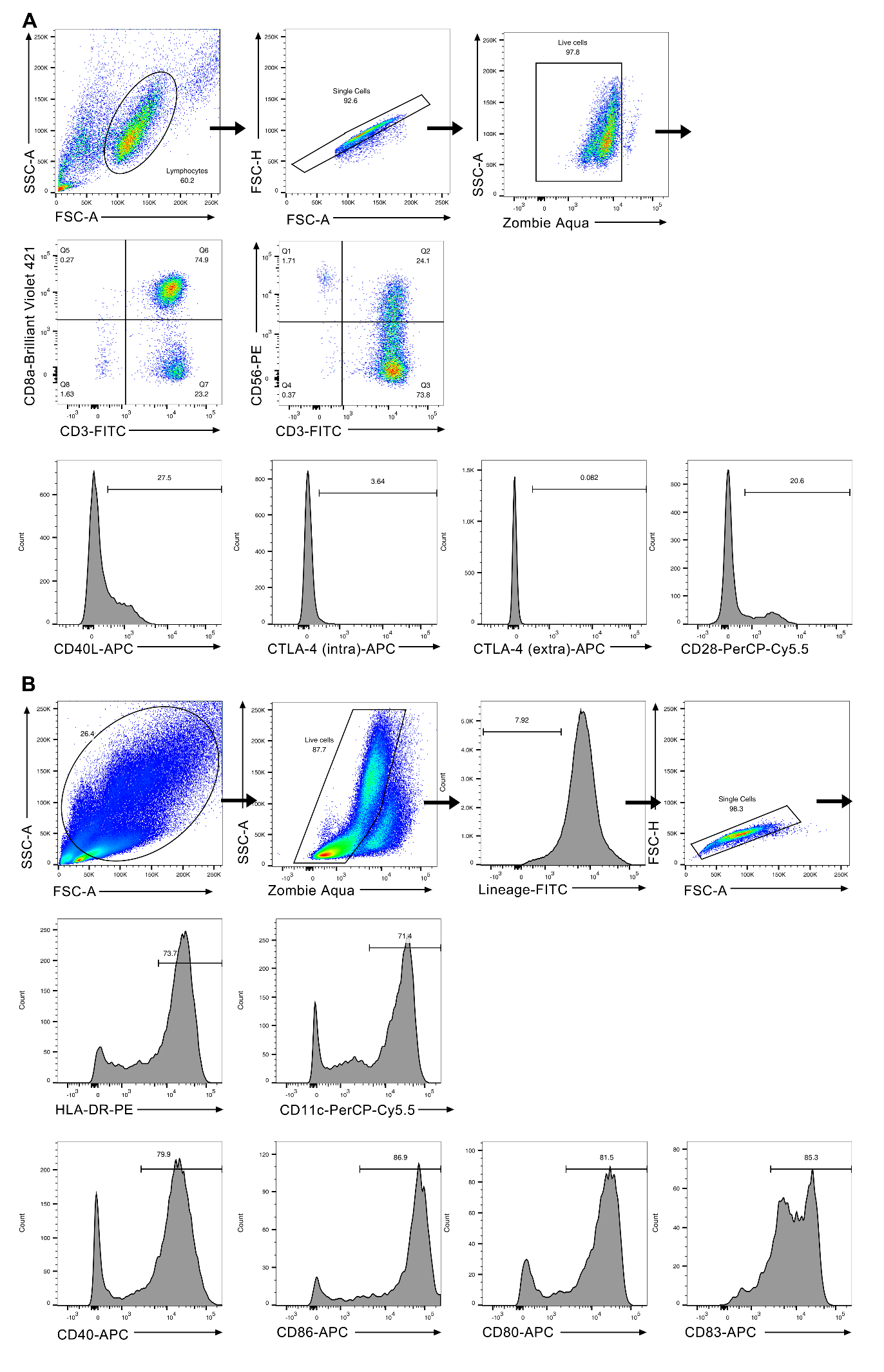
**

**Figure S1** Gating strategy for the phenotyping of DC and CIK cells. **(A)** Gating strategy of CIK cells. Zombie Aqua was used to exclude dead cells. CIK cells on day 14 was shown as a representative. **(B)** Gating strategy of DCs. Lineage 1 cocktail (CD3, CD14, CD16, CD19, CD20 and CD56-FITC)-negative were gated in Lineage-FITC histogram. The gated cells were then analyzed for PE-HLA-DR, PerCP/Cyanine 5.5-CD11c, APC-CD40, APC-CD80, APC-CD83, APC-CD86. DCs on day 9 was shown as a representative.

**
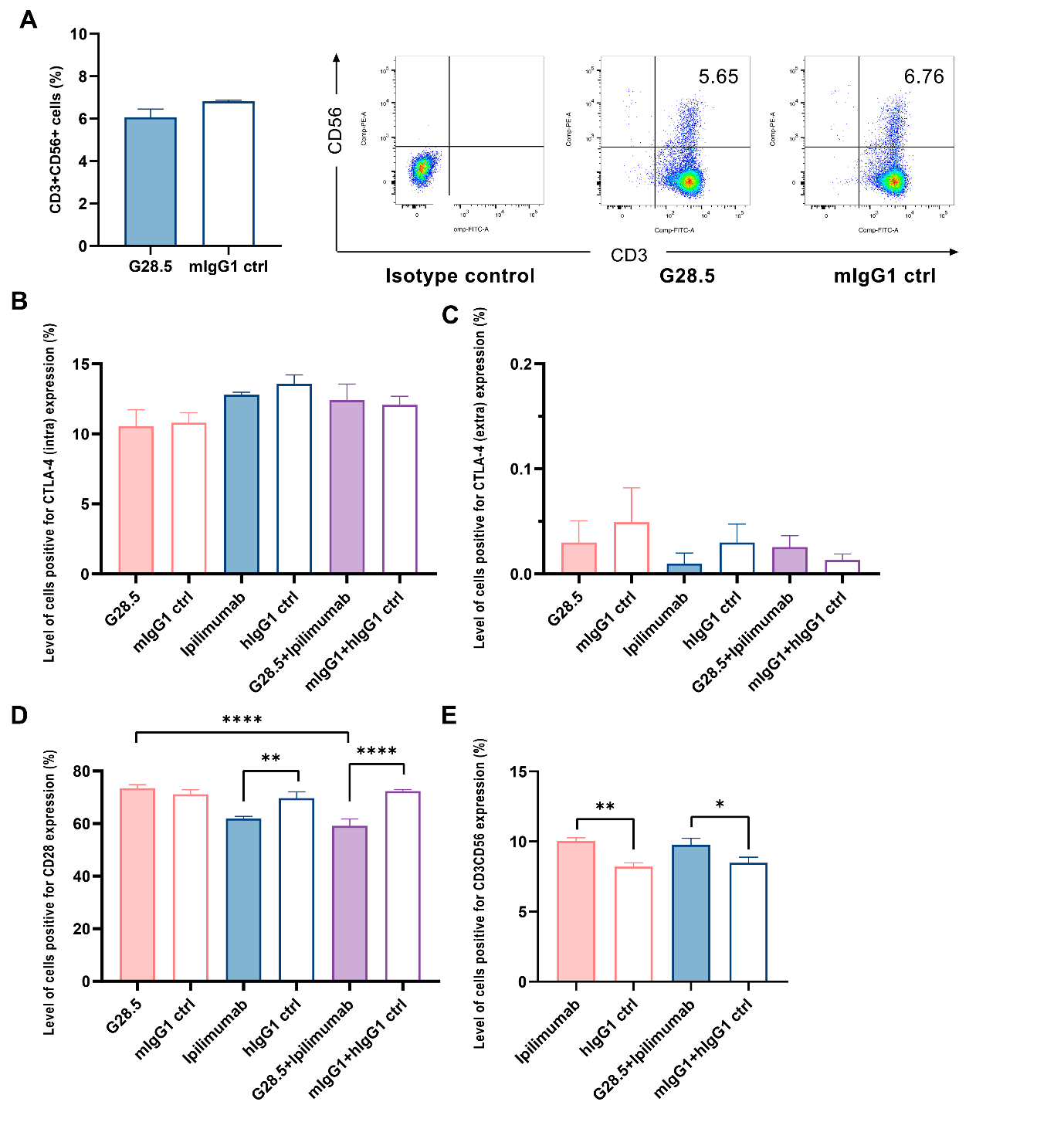
**

**Figure S2** The effect of G28.5 and ipilimumab on the phenotype of CIK cells. **(A)** G28.5 had no direct effect on the CD3+CD56+ population of CIK cells alone. **(B)** The effect of G28.5 and ipilimumab on intracellular expression of CTLA-4. **(C)** The effect of G28.5 and ipilimumab on extracellular expression of CTLA-4. **(D)** The effect of G28.5 and ipilimumab on the expression of CD28. **(E)** Ipilimumab increased the CD3+CD56+ population of CIK cells. Intra: intracellular; extra: extracellular. (* P < 0.05, ** P < 0.01, **** P < 0.0001)

**
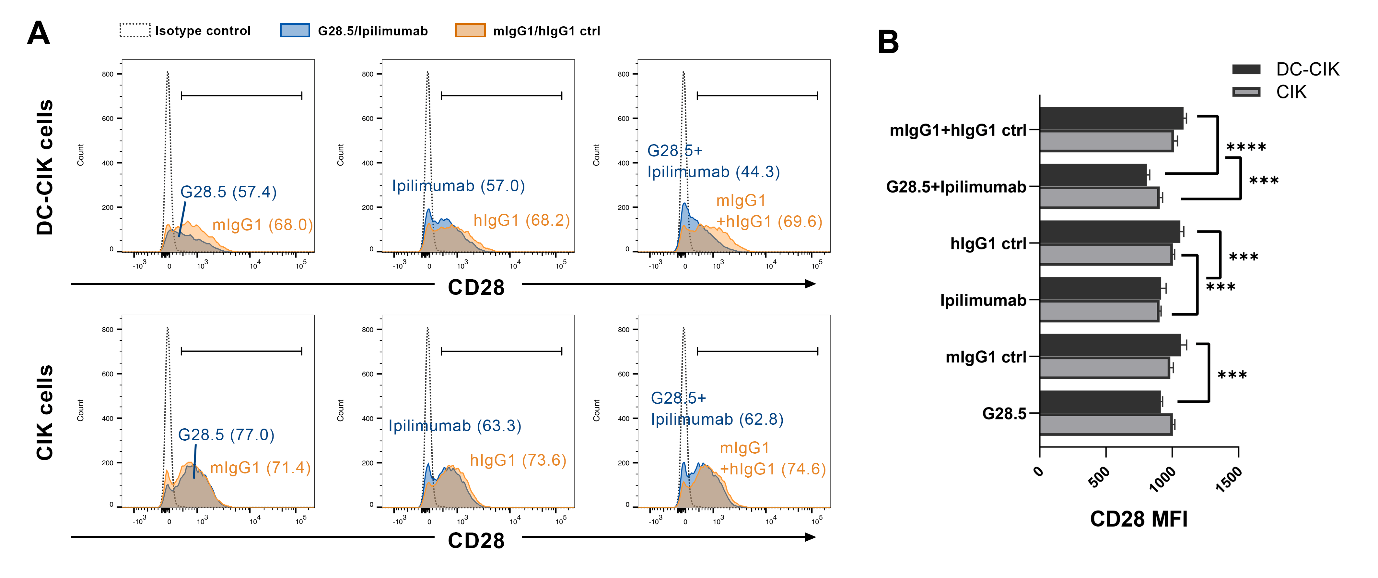
**

**Figure S3** Flow cytometry analysis of CD28 after G28.5 and ipilimumab treatment. **(A)** One representative phenotyping analysis of Figure 3C. Quantification of the MFI is shown in **(B)**. (*** *P* < 0.001, **** *P* < 0.0001)

**
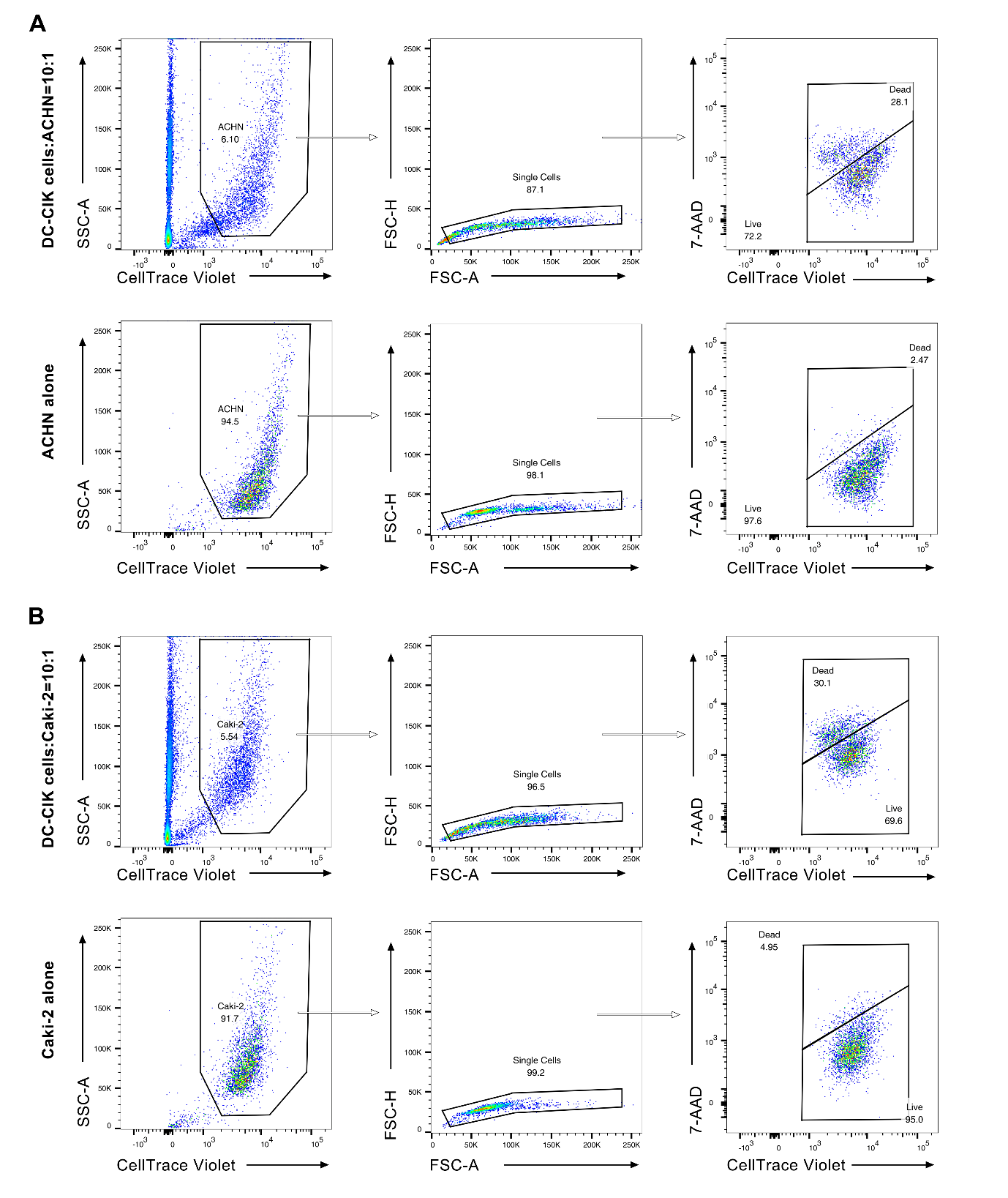
**

**Figure S4** Gating strategy for cytotoxicity of DC-CIK cells against tumor cells. Achn and Caki-2 cells were first labeled with CellTraceViolet to distinguish them from DC-CIK cells. 7-AAD was used to detect dead cells. **(A)** Gating strategy for cytotoxicity of DC-CIK cells against ACHN. **(B)** Gating strategy for cytotoxicity of DC-CIK cells against Caki-2.


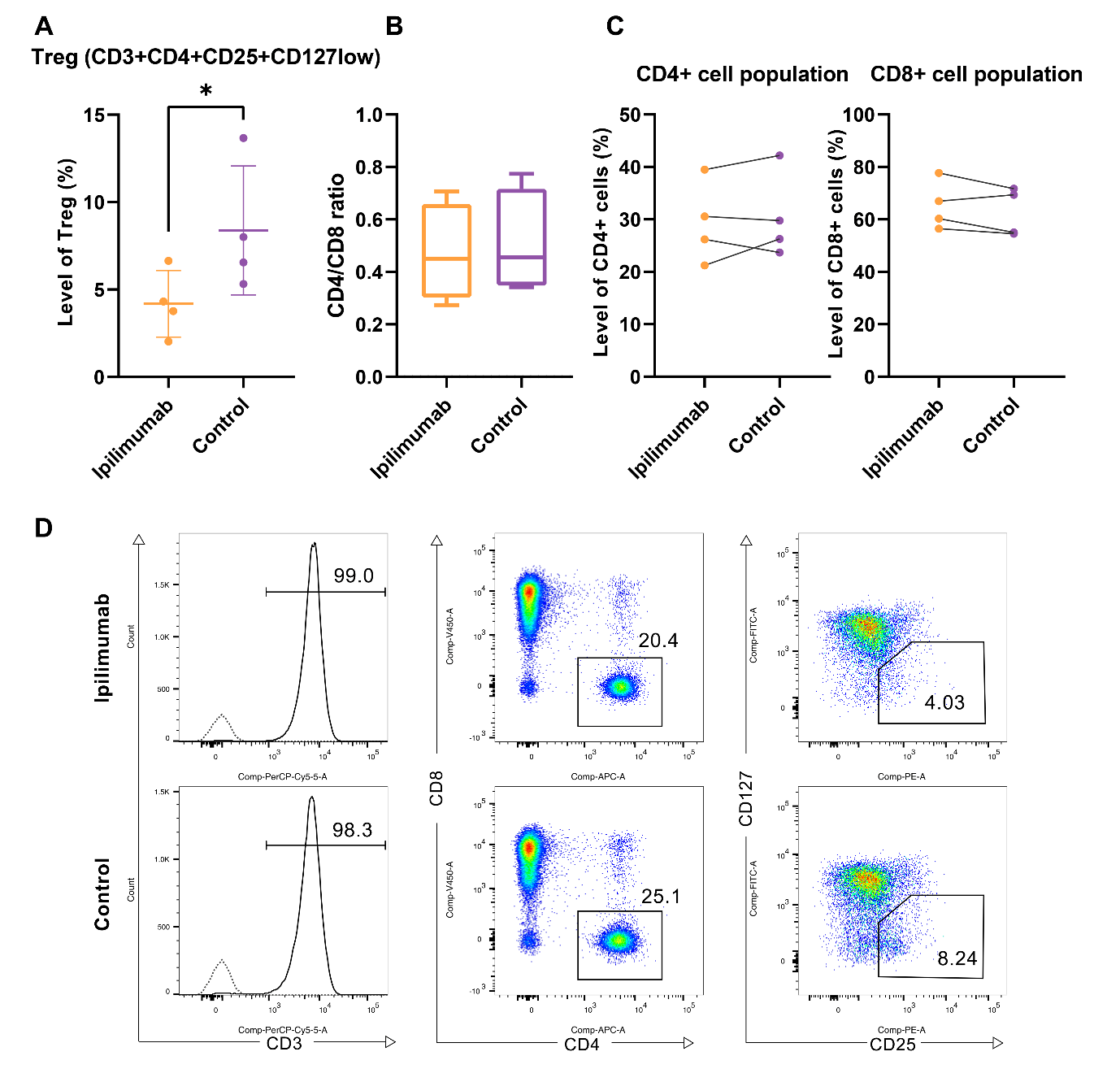


**Figure S5** Ipilimumab decreases the Treg population in activated T cells. **(A)** Treg population in activated T cells after treatment with ipilimumab or control. T cells in peripheral blood mononuclear cells were activated by 20 ng/mL of anti-CD3 monoclonal antibody and expanded with 100 IU/mL of IL-2 according to the protocol of Yano *et al*. (*24*). Activated T cells were incubated with the presence or absence of 10 μg/ml ipilimumab for 14 days. Tregs (CD3+CD4+CD25+CD127low) in activated T cells were detected by flow cytometry. Each bar represents the mean ± SD of four donors. (* *P* < 0.05) **(B)** A slightly decreased CD4/CD8 ratio is shown in activated T cells treated with ipilimumab. **(C)** The proportion of CD4+ (left) and CD8+ (right) cells were detected in the ipilimumab and control groups. **(D)** One representative flow cytometry analysis is shown.
